# Quercetin Induces Apoptosis Through Downregulating P4HA2 and Inhibiting the PI3K/Akt/mTOR Axis in Hepatocellular Carcinoma Cells: An In Vitro Study

**DOI:** 10.1002/cnr2.70220

**Published:** 2025-05-10

**Authors:** Junli Zhang, Jiayi Guo, Ying Qian, Lianchen Yu, Junrao Ma, Biao Gu, Weichun Tang, Yi Li, Hongwei Li, Wenjuan Wu

**Affiliations:** ^1^ The Third People's Hospital of Bengbu Affiliated to Bengbu Medical University Bengbu China; ^2^ Anhui Provincial Key Laboratory of Tumor Evolution and Intelligent Diagnosis and Treatment Bengbu China; ^3^ Bengbu Medical University Key Laboratory of Cancer Research and Clinical Laboratory Diagnosis Bengbu Medical University Bengbu China; ^4^ Department of Biochemistry and Molecular Biology School of Laboratory Medicine, Bengbu Medical University Bengbu China

**Keywords:** apoptosis, hepatocellular carcinoma, P4HA2, quercetin

## Abstract

**Background:**

Quercetin is a natural product with multiple activities, which possesses a promising antitumor effect on malignancies. The involvement of proline 4‐hydroxylase II (P4HA2) in collagen synthesis is crucial in the growth of tumor cells. Apoptosis is a programmed cell death requisite for the stability of the intracellular environment. However, the relationship between quercetin and cell apoptosis, as well as the impact of P4HA2 in this connection, has not yet been specified in hepatocellular carcinoma(HCC).

**Aims:**

The present study used HCC cells to investigate how quercetin regulates P4HA2 and influences cell proliferation and apoptosis.

**Methods and Results:**

The outcomes reveal that quercetin can impede the viability and growth of HCC cells and generate cell apoptosis in a dose‐dependent manner. Additionally, quercetin prompts downregulation of P4HA2, leading to cell apoptosis in HCC cells, and knocking down P4HA2 can enhance this effect. Furthermore, we pretreated HCC cells with inhibitors (Z‐VAD‐FMK, LY294002) or activators (740Y‐P) and found that the PI3K/Akt/mTOR pathway was occupied with quercetin‐induced cell apoptosis.

**Conclusion:**

This investigation reveals that quercetin compels apoptosis in HCC cells by diminishing P4HA2 and restraining the PI3K/Akt/mTOR axis.

## 1 Introduction

There are approximately 900 000 new cases of liver cancer worldwide every year, and its mortality rate is very high, with about 800 000 deaths per year and an increasing trend year by year [1]. Hepatocellular carcinoma is the main type of primary liver cancer. Given the lack of precise clinical symptoms and signs, the initial diagnosis and treatment of HCC is nearly hopeless, and approximately 80% of HCC patients are identified with advanced stage, thus unable to perform surgery [2, 3]. The overall 5‐year survival rate of patients is inferior to 30%, with most having poor prognosis [4, 5]. Advanced HCC patients are often treated with targeted drugs, and lenvatinib is one of the commonly used drugs. However, its clinical application is limited due to the resistance and an incomplete understanding of its resistance mechanism [6, 7, 8]. Hence, it is indispensable to expand novel therapy policies and pursue latest medicine for the treatment of HCC.

Prolyl 4 hydroxylases (P4Hs) are major enzymes that promote collagen biosynthesis. These are α2β2 tetramers located within the endoplasmic reticulum of cells. The biosynthesis of collagen is a multi‐step process; one important key enzyme is P4Hs. It has five subtypes: P4HA1, P4HA2, P4HA3, P4HB, and P4HTM [9, 10]. Due to the low expression level of P4HA2 in the liver under physiological conditions and the significant multiple discrepancies in the molecular level of P4HA2 among cancer and adjacent tissues in HCC compared to P4HA1 [11], the focus is on studying the biological effects and underlying mechanisms caused by abnormally high expression of P4HA2 in HCC [12, 13]. The P4Hs family is elevated in various tumors and is associated with poor patient prognosis [14, 15]. P4HA2 plays a role in prostate cancer metastasis. Yes‐associated protein 1 (YAP1) depends on P4HA2 to activate epithelial‐mesenchymal transition (EMT) to make tumor cells metastasize [16, 17]. In addition, P4HA2 can also enhance the glycolysis of cervical cancer cells to accelerate the tumor process [18]. Bioinformatics analysis exposed that P4HA2 was occupied with the regulatory mechanism of cervical cancer and was connected with shorter overall survival (OS) and recurrence‐free survival (RFS) [19]. In HCC, the research scope of P4HA2 is relatively limited, which is related to the effects caused by its downstream end product collagen. Aspirin regulates NF‐κB pathways to inhibit P4HA2 protein activity, preventing collagen deposition and inhibiting tumor growth [20]. P4HA2 is specifically correlated with hepatitis B virus X protein (HBx). HBx can maintain P4H by targeting P4HA2 mRNA in HCC [21]. Studies have found that P4HA2 is also vital in tumor drug resistance. Hypoxia‐inducible factor‐1 (HIF‐1) can directly bind to the P4HA2 promoter under hypoxic conditions, strengthen its stability, and enhance the resistance of bladder cancer to Erdafitinib. At the same time, P4HA2 is the key to Erdafitinib resistance [22]. Another study showed that the transcription factor CEBPB promoted the development of isocitrate dehydrogenase 1 wildtype (IDH1 wt) gliomas by upregulating P4HA2 protein in glioma cells at the transcriptional level [23]. In addition, P4HA2 is also involved in the resistance to temozolomide, the primary therapeutic drug for glioma [23]. Therefore, P4HA2 is considered an oncogene, and its inactivation may be a valuable strategy for treating liver cancer.

Quercetin (Que; 3, 5, 7, 30, 40 pentahydroxyflavones) is a type of pentahydroxyflavonoid compound, primarily discovered in diverse plants similar to apples, cherries, broccoli, and onions, along with in dietary items like green tea and red wine [24, 25, 26, 27, 28]. It is documented that quercetin has an assortment of biological effects, involving antioxidant, anti‐cancer, anti‐inflammatory, and anti‐diabetes [29, 30, 31, 32, 33]. Quercetin, a flavonoid compound, shows significant possibility in oncology as a result of its chemopreventive property in cell and animal models [34]. Most traditional Chinese medicine ingredients provoke biphasic and dose‐dependent effects [35, 36]. For example, phyllanthin not only has a protective effect against diethylnitrosamine (DEN) induced liver carcinoma in Wistar Albino rats, but also has anti‐tumor potential against human hepatocellular carcinoma HepG2 cells [37]. Similarly, quercetin also has similar effects. At a lower level, quercetin functions as an antioxidant, thereby causing protective function, but at a higher level, quercetin serves as a prooxidant, producing a chemotherapeutic impact [24, 38, 39]. Quercetin exhibits good anticancer activity in both monotherapy and combination chemotherapy drugs. Combined quercetin/sorafenib treatment markedly improved the morphology of the induced liver damage and showed significant antioxidant and anti‐tumor effects [40, 41]. Experiments have proved that quercetin can significantly impede the apoptosis process in a wide array of malignant tumors, for instance, breast cancer [31, 34], human non‐small cell lung cancer [30], prostate cancer [29, 30], esophageal squamous cell carcinoma [42], and so on. The mechanism of action is also different for different types of tumor cells. Quercetin reverses docetaxel resistance through the PI3K/Akt signaling pathway in prostate cancer cells, leading to apoptosis promotion [43]. Quercetin inhibits lung cancer cell viability dose‐dependently and induces mitochondria‐dependent apoptosis, which involves the SIRT1/AMPK signaling pathway [44]. Furthermore, the anti‐tumor influence of quercetin may be closely linked to quercetin‐mediated lysosomal activation and ferritin degradation [45]. Whether quercetin can induce apoptosis in hepatoma cells through signaling pathways remains unreported.

Previous studies of our research group confirmed that P4HA2 affects the growth, migration, and invasion of HCC cells via stimulating the PI3K/Akt/mTOR pathway [46]. Nonetheless, its biological effects on quercetin and P4HA2 remain largely unclear here. This study displayed that quercetin restrains the cells proliferation and leads to apoptosis in HCC. Quercetin inhibits the PI3K/Akt/mTOR pathway by downregulating P4HA2. This survey aspires to reveal the mechanism of quercetin over the HCC cell proliferation and apoptosis and present new notions for the molecular therapeutics of HCC.

## 2 Methods and Materials

### 2.1 The Culturing of HCC Cells

Two human HCC cell lines, SNU‐449 and Hep‐3B (CAS, Shanghai, China), were cultured in RPMI‐1640 (SNU‐449) or Dulbecco's Modified Eagle's Medium (DMEM) (Hep‐3B) with 10% FBS (fetal bovine serum) (Gibco, USA) in a cell incubator (37°C, 5% CO_2_).

### 2.2 Reagents and Antibody

Quercetin was from MCE (CAS: HY‐18085). LY294002, 740Y‐P, and Z‐VAD‐FMK also were brought from MCE (Shanghai, China). Antibodies were purchased as follows: Primary antibodies against Bcl‐2 (1:2000), Bax (1:2000), Caspase‐9 (1:100), PARP (1:3000), P4HA2(1:1000), and β‐actin (1:5000) were obtained from Proteintech (Chicago, IL). p‐PI3K (1:1000), PI3K (1:1000), Akt (1:1000), p‐Akt (1:2000), P‐mTOR (1:1000), and mTOR (1:2000) were obtained from Cell Signaling Technology (Danvers, MA, USA). The secondary antibodies (1:5000) were purchased from Abcam (UK).

### 2.3 Cell Proliferation Assay

Collect cells (1000 cells/well), inoculate them with 96 well plates, and treat them with quercetin at indicated concentrations or times (0, 3.125, 6,25, 12.5, 25, 50, 100, and 200 μM). Then, add 10 μL reagent solutions into HCC at 37°C for 2 h. Finally, measure the absorbance.

### 2.4 Colony Formation Assay

Inoculate approximately 500 cells into each well of a 60 mm culture dish and continue culturing in the incubator until cell clones appear. Then, fix the cells and stain with crystal violet for 30 min. Visible colonies were counted and pictured.

### 2.5 Ethynyl‐2′‐Deoxyuridine (EdU)‐based Proliferation Assay

The cell was handled with quercetin for 48 h and collected. Cell proliferation was measured using an EdU kit. All fluorescent images were investigated employing a Zeiss fluorescence microscope.

### 2.6 TUNEL Assay

One‐step TUNEL staining measured the apoptosis of cells after 48 h of treatment with quercetin. In short, after treatment with cell crawling, 4% paraformaldehyde solution fixation, and 0.3% Triton x‐100 permeability, HCC cells were tagged with fluorescein‐12‐dUTP, and the nuclei were spotted with 4,6‐diamino‐2‐phenylindole (DAP). Take photographs utilizing a Zeiss fluorescence microscope.

### 2.7 Flow Cytometry Assay

HCC cells were treated with or without quercetin for 48 h and were harvested by centrifugation at 1000 revolutions/min for 5 min. Afterwards, the collected cells were stained with Annexin‐V/PI in the dark for 30 min. The apoptosis rate of the SNU‐449 and Hep‐3B cells was determined using a flow cytometer (Cytomics FC500).

### 2.8 RNA Extraction and Quantitative Real‐Time PCR

HCC cells were isolated from total RNA according to the Trizol protocol (Invitrogen). Complementary DNA (cDNA) was synthesized using SuperScript III RNase H Reverse Transcriptase (Thermo Fisher Scientific, America). SYBR premixex taq (TAKARA, China) was used to amplify a LightCycler 96 system (Roche, America). The polymerase chain reaction was carried out in triplicates. RNA expression was normalized to that of β‐actin expression and quantified by the 2^−^△△Ct method. Primers P4HA2 and β‐actin have lengths of 24 and 20 bp, respectively. The primers were as follows: P4HA2, 5'‐GGCCTGGTTTGGTGTCCTG3' and 5'‐GCCCAGCTCTTAATCTTGGAAAG3' β‐actin, 5'‐GTGGACATCCGCAAAGAC‐3' and 5'‐AAAGGGTGTAACGCAACTA‐3'.

### 2.9 Protein Extraction and Western Blotting

Cells in each group were collected, and total proteins were extracted using RIPA buffer (Beyotime, China) and denatured at 95°C. The 40 μg of BCA Kit quantified protein per well was subjected to electrophoresis and membrane transfer. Subsequently, the membranes were blocked and incubated with the primary antibodies against P4HA2, Bcl‐2, Bax, Caspase‐9, PARP, p‐PI3K, PI3K, Akt, p‐Akt, P‐mTOR, and mTOR, and β‐actin overnight at 4°C. The next day, the second antibody for P4HA2 and β‐actin was incubated for 1 h. Finally, the gel imaging system was exposed.

### 2.10 Statistical Analysis

All experimental data in the study are presented as the mean ± SD. One‐way analysis of variance (ANOVA) followed by Dunnett's post hoc test was used for multiple group comparisons using SPSS 19.0 statistical software. Statistical significance was set at *p* < 0.05.

## 3 Result

### 3.1 Quercetin Inhibited Viability and Proliferation of Human HCC Cells

**FIGURE 1:**
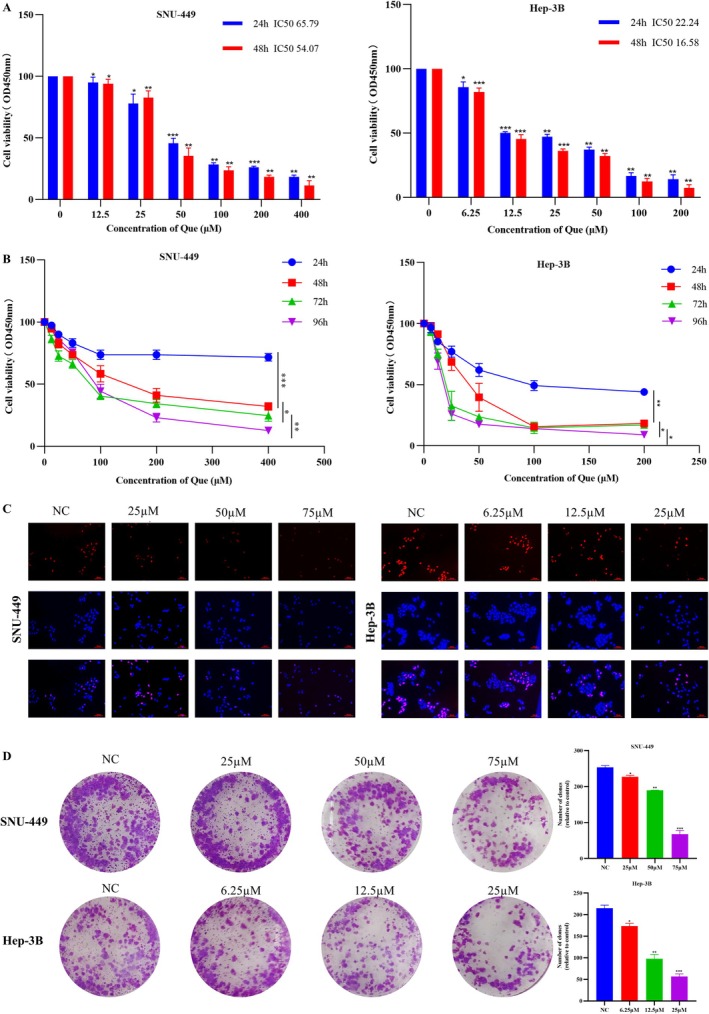
Quercetin inhibited viability, and proliferation of human HCC cells. (A) SNU‐449 and Hep‐3B cells were treated with quercetin of various concentrations for 24 h and 48 h, and cell counting kit‐8 (CCK‐8) assay was performed to detect the viability of these two cell lines. (B) The Effects of quercetin on the viability of SNU‐449 and Hep‐3B cells for 24, 48, 72 and 96 h, were measured using the CCK‐8 assay. (C) The effect of quercetin on SNU‐449 and Hep‐3B cell proliferation was tested by EdU incorporation assay. HCC cells were treated with quercetin for 48 h. (D) Colony formation assay was also performed to explore the effect of quercetin on cell proliferative ability. Analysis of variance test; *p < 0.05, **p < 0.01, ***p < 0.001 versus the control.

To investigate whether quercetin can inhibit the HCC cell viability and proliferation. SNU‐449 and Hep‐3B cell lines were pretreated with quercetin of different concentrations (0, 12.5, 25, 50, 100, 200, and 400 μM) (0, 6.25, 12.5, 25, 50, 100, and 200 μM), respectively. The cell viability was observed at 24 and 48 h by CCK‐8 assay, and the results showed that quercetin can dose‐dependently inhibit the activity of SNU449 and Hep‐3B cells (Figure 1A). After 48 h of quercetin treatment, the IC50 values of SNU449 and Hep‐3B cells were 54.07 μM and 16.58 μM, respectively. Moreover, the repressive impact of quercetin on SNU449 and Hep‐3B cell lines at different treated times (24, 48, 72, and 96 h) was detected. Results indicated that quercetin significantly lessened cell viabilities in a time‐ and dose‐dependent manner (Figure 1B). This experiment selected 25 μM, 50 μM and 75 μM quercetin for treating SNU‐449 cells, and 6.25 μM, 12.5 μM and 25 μM quercetin for Hep‐3B cells. The colony formation assay confirmed the dose‐dependent suppression of proliferation by quercetin (Figure 1C). This phenomenon was confirmed by EdU incorporation assay with quercetin at 48 h (Figure 1D).

### 3.2 Quercetin Induced Apoptosis by Inhibiting the PI3K/Akt/mTOR Signaling Pathway

**FIGURE 2:**
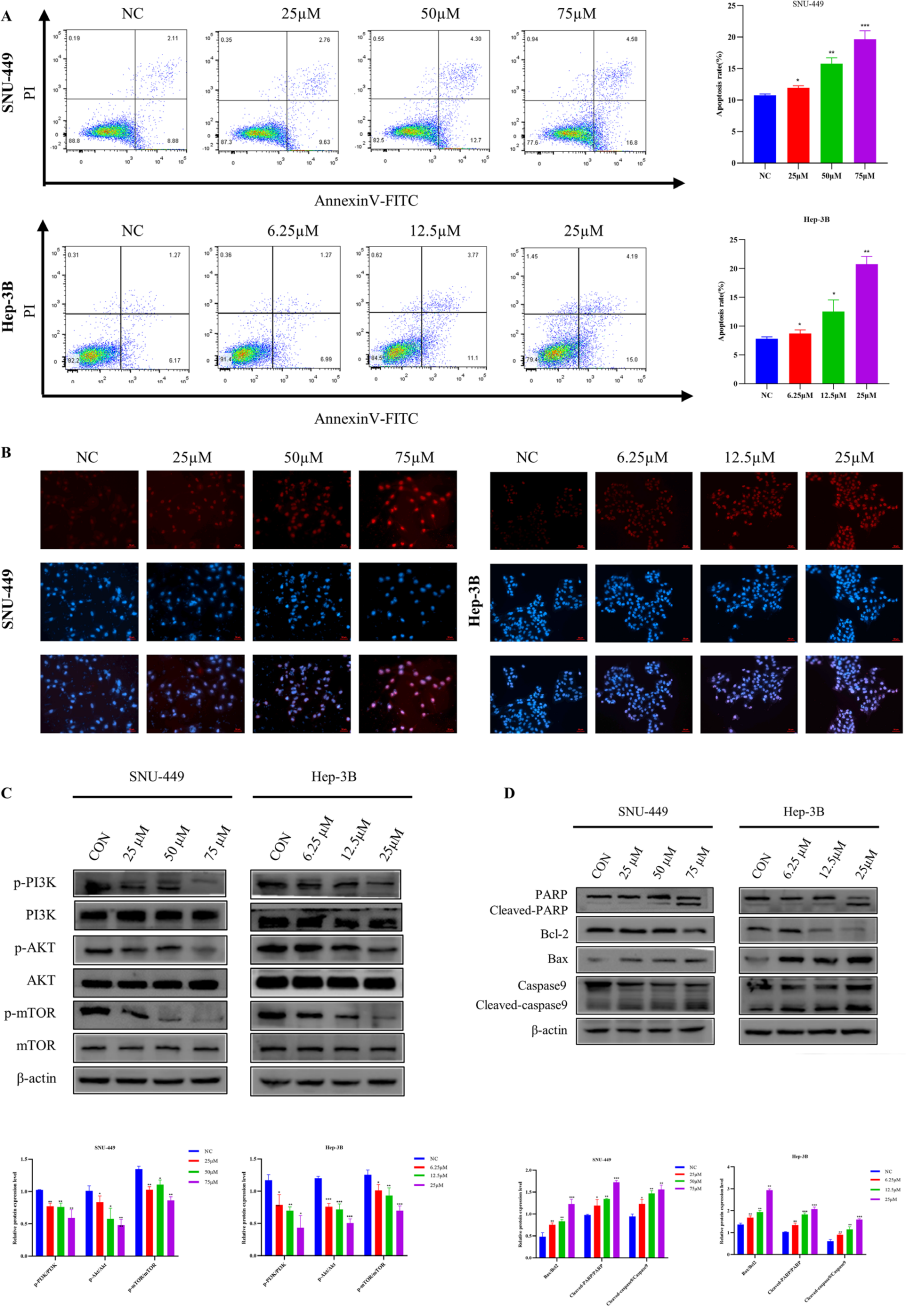
Quercetin induced apoptosis by inhibiting the PI3K/Akt/mTOR signaling pathway in SNU‐449 and Hep‐3B cells. (A) The apoptosis assays of SNU‐449 and Hep‐3B cells incubated by quercetin. (B) TUNEL staining(x20) of SNU‐449 and Hep‐3B cells exposed to quercetin for 48 h. Representative images show abnormal or apoptotic cells (Red fluorescence). (C) Cells were incubated with various concentrations (0, 25, 50 and 75 μM) or (0, 6.25, 12.5 and 25 μM) of quercetin for 48 h. p‐PI3K, p‐Akt, and p‐mTOR expression levels were analyzed by western blot. (D) The expression of Bax, Bcl2, cleaved caspase‐9, and cleaved caspase‐PARP proteins in apoptosis pathway were measured. Data were expressed as the mean ± SD from at least three independent experiments. Analysis of variance test, *p < 0.05, **p < 0.01, ***p < 0.001 versus the respective control.

For the purpose of investigating the potential mechanism of quercetin around exerting anti‐cancer effects, HCC cells were pretreated with several concentrations of quercetin and then double‐stained with FITC and PI. The cell apoptosis rate was significantly higher than in untreated cells and showed a dose‐dependent relationship (Figure 2A). Quercetin significantly induced late‐stage apoptosis at concentrations of 75 μM and 25 μM. Furthermore, TUNEL staining found that the quantity of positive cells gradually rose along with the concentration of quercetin (Figure 2B), implying the primary character of apoptosis in the anti‐cancer impact of quercetin. Previous studies have shown that proteins in crucial signaling pathways can regulate tumor growth [47]. This study speculates that quercetin may regulate cell proliferation and apoptosis via the PI3K/Akt/mTOR axis. The outcomes displayed that quercetin abbreviated the expression of p‐PI3K, p‐Akt, and p‐mTOR proteins in SNU‐449 and Hep‐3B cells compared to control cells, and this discount was dose‐dependent. In contrast, it had little impact on the total PI3K, AKT, and mTOR (Figure 2C). Additionally, apoptosis‐related proteins Bax, cleaved caspase‐9, and cleaved caspase‐PARP increased dose‐dependently (Figure 2D). However, the anti‐apoptosis protein, Bcl‐2, declined with the treatment of quercetin (Figure 2D).

### 3.3 Quercetin Exerts Anti‐Tumor Effects by Regulating P4HA2

**FIGURE 3:**
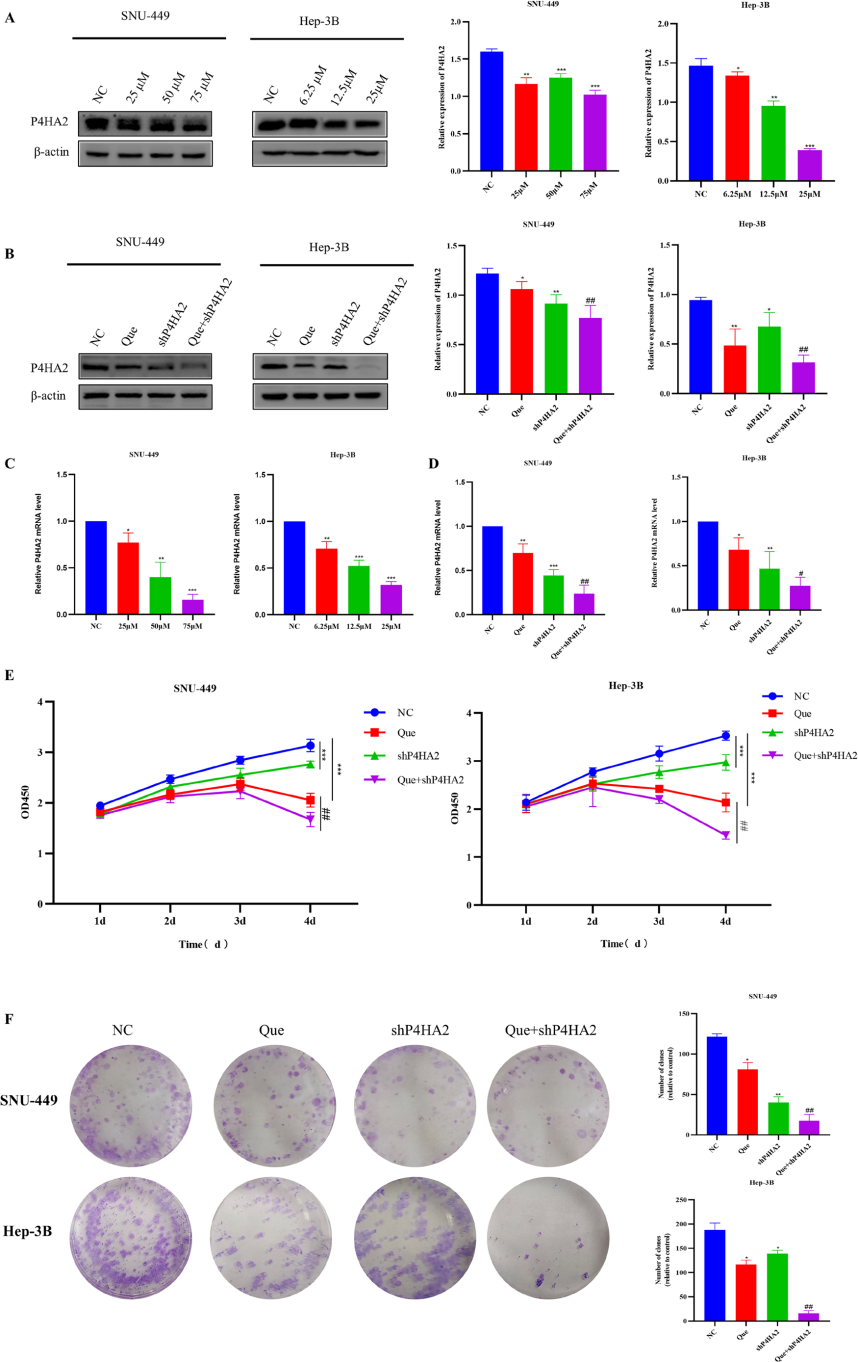
Quercetin exerts anti‐tumor effects by regulating P4HA2 in HCC cells. (A) Western blotting analysis of P4HA2 in SNU‐449 and Hep‐3B cells treated with quercetin for 48 h. The gray values were calculated. (C) RT‐PCR analysis of the effects of quercetin on mRNA expression of P4HA2 in HCC cells. The data are shown as the mean ± SD. (B) P4HA2 expression was tested by immunoblotting in HCC cells with shP4HA2 lentivirus vector transfection plus quercetin treatment. (D) The expression of P4HA2 mRNA was detected by RT‐PCR. The data are expressed as the mean ± SD. (E) The colony‐formation assay was used to evaluate cell growth in SNU‐449 and Hep‐3B. Analysis of variance test; *p < 0.05, **p < 0.01, ***p < 0.001 versus NC, #*p* < 0.05, ##*p* < 0.01, ###*p* < 0.001 for Que + shP4HA2 versus Que.

Based on past research by the group, it was discovered that P4HA2 is at a high level in HCC cells. P4HA2 impressed the proliferation and metastasis of HCC cells via changing the PI3K/Akt/mTOR axis, expanding the occurrence and development of HCC [46]. This survey researched whether quercetin regulates the P4HA2 levels to inhibit cell viability in HCC. Our evidence shows that quercetin restrained the expression of P4HA2 at molecular level (Figure 3A,C). P4HA2 suppression might be a cause for the quercetin‐mediated inhibition of cell viability. To clarify the possible character of P4HA2, this experiment reduced P4HA2 expression by transfection with the lentivirus vector shP4HA2 and co‐treated HCC cells with quercetin, successfully diminishing the P4HA2 level (Figure 3B,D). Quercetin, along with shP4HA2 transfection, lessened the P4HA2 level to a lower level than that with a single quercetin or shP4HA2 treatment only (Figure 3B,D). The CCK8 data show that down‐regulation of P4HA2 reduces cell viability (Figure 3E). Combining quercetin and shP4HA2 resulted in a greater decrease in HCC cell viability than single therapy (Figure 3E). Then, the clone formation survival experiments and EdU experiments were conducted to detect the proliferation effect of each group of cells. The consequences showed that after quercetin treatment, the cell proliferation ability of the Que + shP4HA2 group was remarkably lower than that of the Que group and the shP4HA2 group (Figures 3F and 5A).

### 3.4 Quercetin Regulates P4HA2 Inducing Cell Apoptosis

**FIGURE 4:**
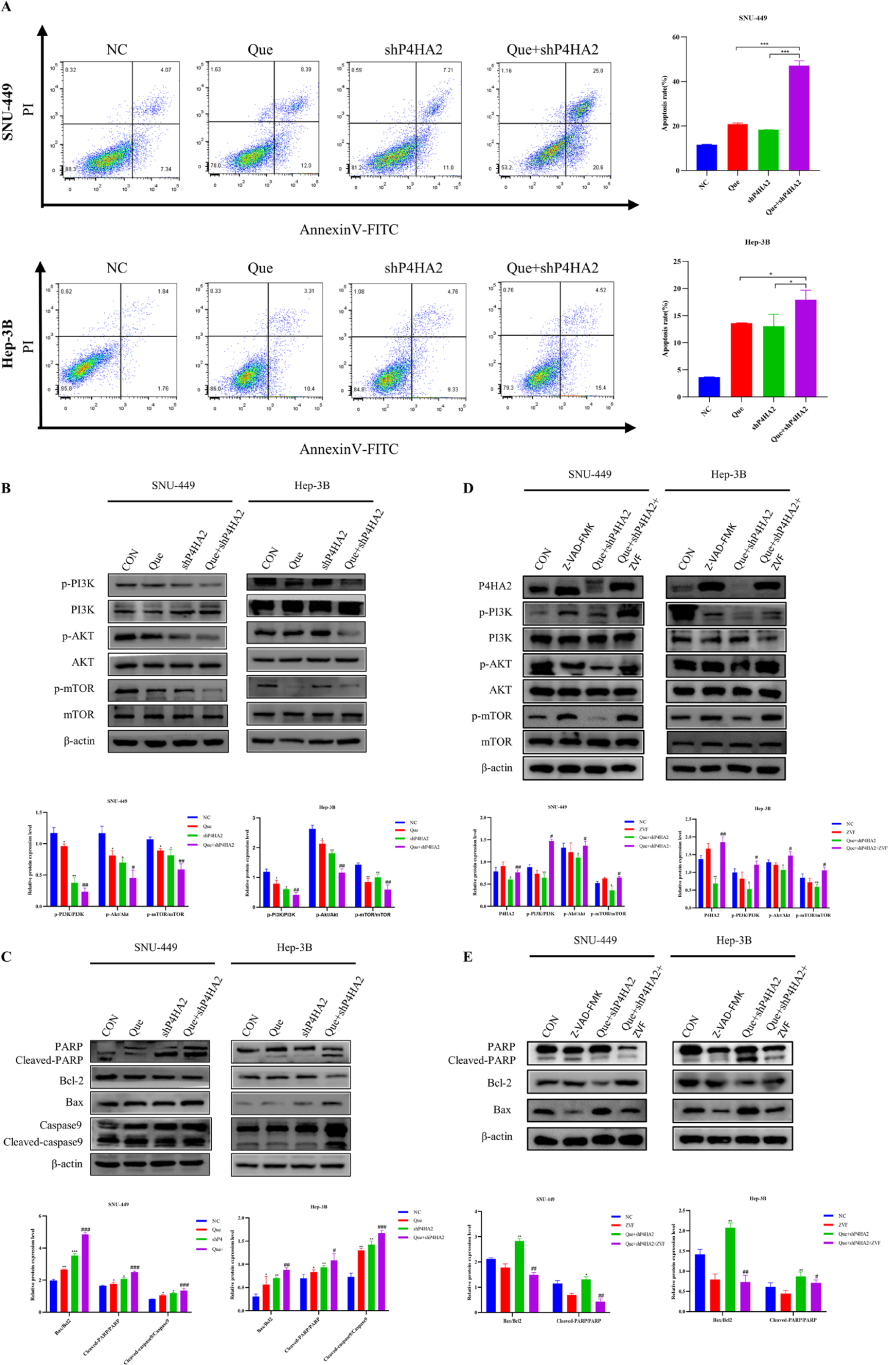
Quercetin regulates P4HA2 inducing cell apoptosis. (A) Detection of apoptosis rate of SNU‐449 and Hep‐3B cells via Annexin V‐FITC/PI double staining. All above data are mean ± SD from average of three experiments. (B, D) Western blotting assay showing the expression of pathways and apoptosis related proteins in the indicated cells. β‐Actin was used as the loading control. (C, E) Protein expression was determined. Cells were pretreated with ZVF (40 μM) for 24 h and then incubated with Que + shP4HA2 group cells (50 or 12.5 μM) for 24 h. *p < 0.05, **p < 0.01, ***p < 0.001 versus the respective control. Analysis of variance test; #*p* < 0.05, ##*p* < 0.01, ###*p* < 0.001 for Que + shP4HA2 versus Que.

**FIGURE 5:**
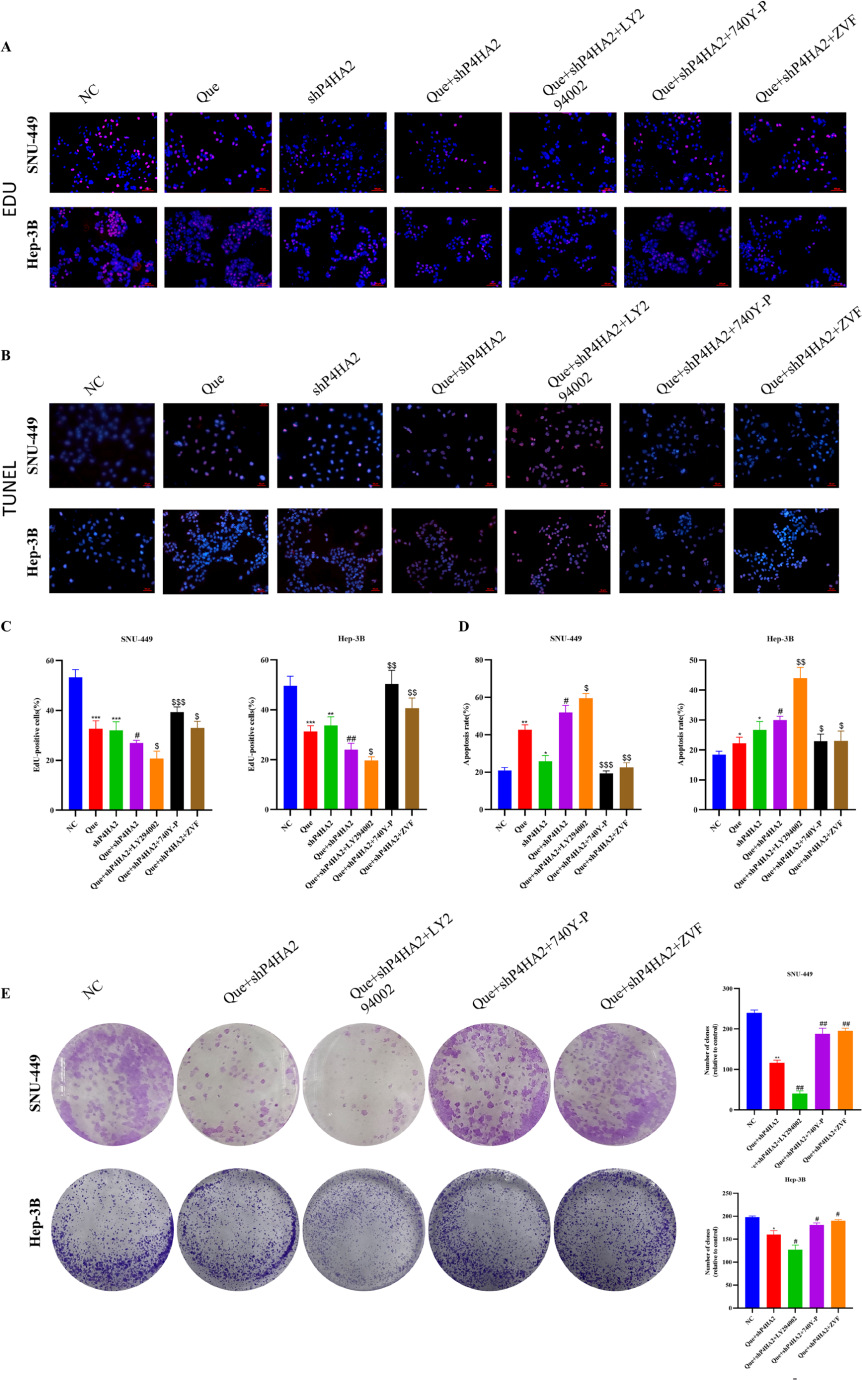
Quercetin‐induced apoptosis by downregulating P4HA2 and inhibiting the PI3K/Akt/mTOR pathway. SNU‐449 and Hep‐3B cells were pretreated with LY294002 (25 μM) or 740Y‐P (30 μM) or ZVF (40 μM) for 24 h, then were incubated with control medium or Que + shP4HA2 (50 or 12.5 μM) for another 24 h. (A, C) EdU stain and colony formation assay(E) was performed to detect the cell proliferation. (B, D) Cell death was examined by TUNEL stained assay. The data are expressed as the mean ± SD. Analysis of variance test, *p < 0.05 for Que or shP4HA2 versus NC, #*p* < 0.05 for Que + shP4HA2 versus Que, $*p* < 0.05 for Que + shP4HA2 + LY294002 or Que + shP4HA2 + 740Y‐P or Que + shP4HA2 + ZVF versus Que + shP4HA2.

Annexin FITC/PI staining was employed to quantify the number of apoptotic cells further. The Que + shP4HA2 group exhibited a meaningful increase in cell apoptosis rate in contrast to the quercetin and shP4HA2 group. The early and late apoptosis rates of the Que + shP4HA2 group were 45.6% and 19.92%, respectively, which were notably greater than those of other groups: the control group (11.41% and 3.7%), the quercetin group (20.39% and 13.71%), and the shP4HA2 group (18.21% and 14.09%) (Figure 4A). Additionally, TUNEL staining results aligned with the aforementioned findings (Figure 5B). To further explore the mechanism by which quercetin downregulates P4HA2 to promote cell apoptosis, the western blot was employed to observe the PI3K/Akt/mTOR pathway proteins and apoptosis‐related proteins. As shown in Figure 4B,C, quercetin treatment of shP4HA2 cells considerably curbed p‐PI3K, p‐Akt, and p‐mTOR proteins in SNU‐449 and Hep‐3B cells. Quercetin augmented the protein level of Bax, cleaved‐caspase9, and cleaved‐PARP while decreasing Bcl2 expression. In the Que + shP4HA2 group, the impact of quercetin on cell apoptosis was intensified.

Z‐VAD‐FMK (ZVF) may inhibit cell apoptosis and be used to verify whether quercetin induces cell apoptosis via inhibiting the level of P4HA2. In comparison to the Que + shP4HA2 group, western blot results presented that ZVF combined with quercetin treatment of shP4HA2 cells appreciably decreased Bax protein level and enhanced the level of Bcl2 (Figure 4E). In addition, the EdU and clone formation assay showed that when quercetin+ShP4HA2 group was combined with ZVF, the promoting consequence of quercetin on cell apoptosis was attenuated (Figure 5A). The TUNEL stain found that the treatment of shP4HA2 cells with ZVF combined with quercetin can prevent the rise of apoptotic cells (Figure 5B). These results offer compelling evidence that the inducing‐apoptosis effects of quercetin are closely linked to P4HA2 and exert their influence by inhibiting the level of P4HA2.

### 3.5 Quercetin‐Induced Apoptosis by Downregulating P4HA2 and Inhibiting the PI3K/Akt/mTOR Pathway

**FIGURE 6:**
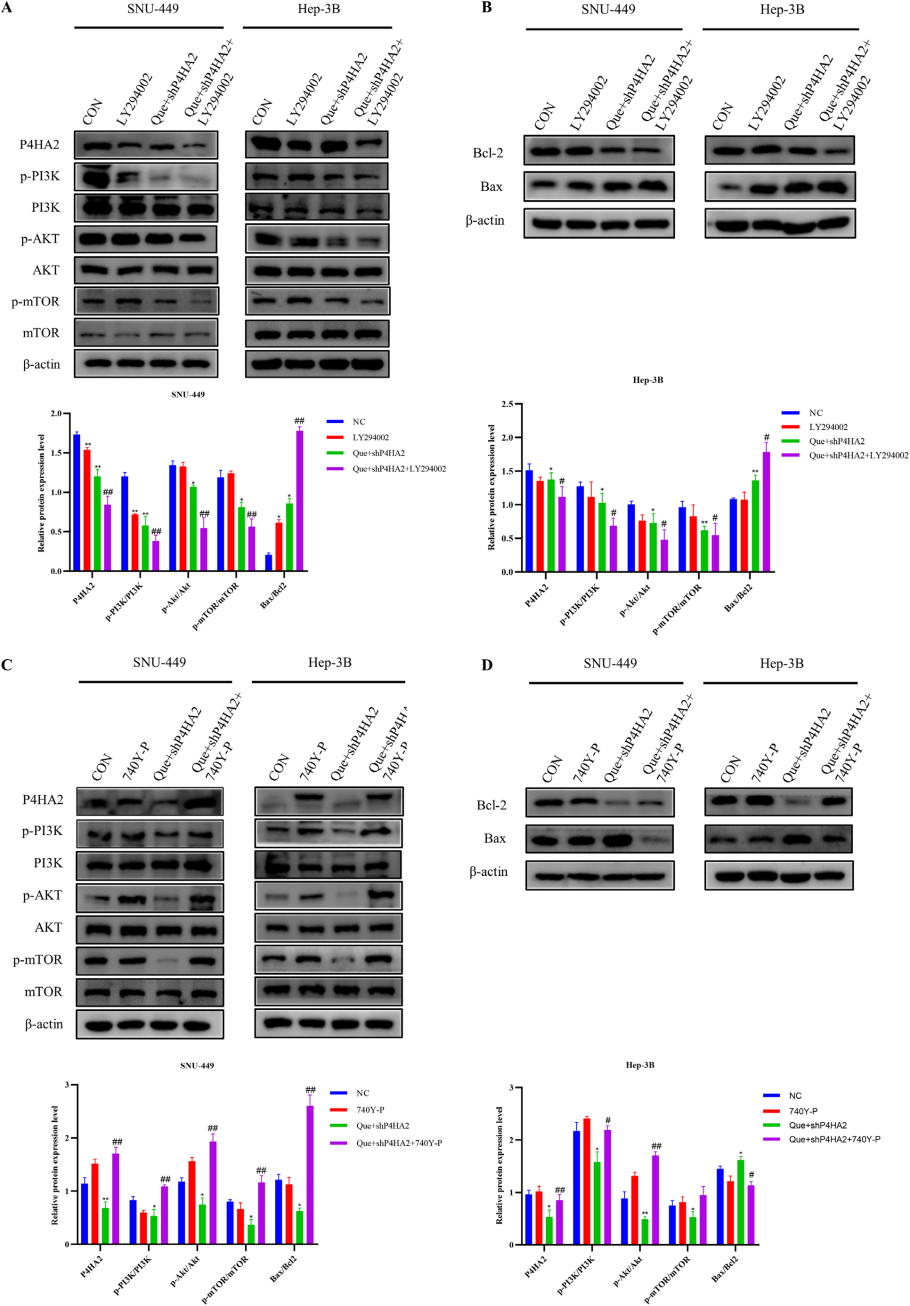
Quercetin‐induced apoptosis by downregulating P4HA2 and inhibiting the PI3K/Akt/mTOR pathway. (A–D) SNU‐449 and Hep‐3B cells were pretreated with LY294002 (25 μM) or 740Y‐P (30 μM) for 24 h, then were incubated with control medium or Que + shP4HA2 (50 or 12.5 μM) for another 24 h. Western blot was performed to detect the expression levels of apoptosis related proteins and pathway proteins. Analysis of variance test; *p < 0.05 for Que or shP4HA2 versus NC, #*p* < 0.05 for Que + shP4HA2 versus Que, $*p* < 0.05 for Que + shP4HA2 + LY294002 or Que + shP4HA2 + 740Y‐P versus Que + shP4HA2.

**FIGURE 7:**
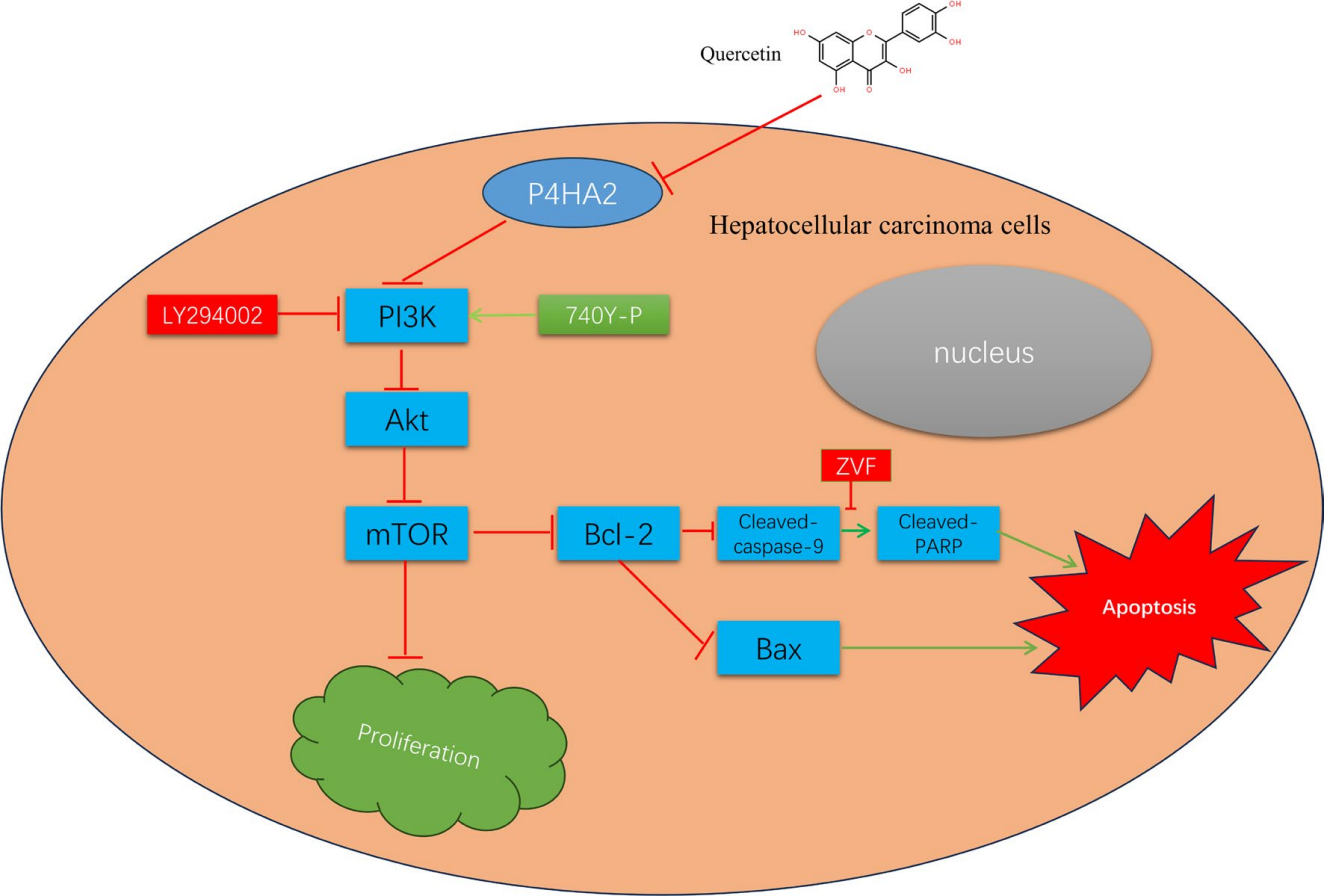
A working model for the mechanism of quercetin on HCC. Quercetin induces caspase‐dependent cell apoptosis and inhibits the proliferation of HCC cells. Furthermore, Quercetin can significantly reduce the expression of P4HA2, and downregulating P4HA2 expression significantly enhances the anti‐tumor effect of quercetin. In addition, inhibition of the PI3K/Akt/mTOR pathway plays a crucial role in quercetin‐induced apoptosis. In the upstream, quercetin‐induced upregulation of P4HA2 inhibits the activation of the PI3K/Akt/mTOR pathway. Altogether, quercetin induces HCC cell apoptosis by downregulating P4HA2 and inhibiting PI3K/Akt/mTOR, suggesting that Quercetin has excellent potential as a new strategy for treating HCC.

The PI3K/Akt/mTOR axis is of great importance in regulating the cell cycle, proliferation, apoptosis, and autophagy. To inspect the part of this pathway in quercetin‐induced cell apoptosis and whether P4HA2 is engaged in quercetin‐induced cell apoptosis. This experiment chose the PI3K inhibitor LY294002 or the PI3K activator 740Y‐P to treat SNU‐449 and Hep‐3B cells. The outcomes indicated that pretreatment with LY294002 meaningfully enhanced the downregulation of p‐PI3K, p‐Akt, and p‐mTOR induced by quercetin‐treated shP4HA2 cells (Figure 6A). Compared to shP4HA2 cells treated with quercetin alone, LY294002 can enhance the inhibitory effect of quercetin on P4HA2, and 740Y‐P can rescue quercetin‐inhibitor P4HA2 levels (Figure 6A,C). In addition, ZVF combined with quercetin treatment of shP4HA2 cells, ZVF rescued the low levels of pathway proteins due to quercetin combined with shP4HA2 cells (Figure 4D). Pretreated cells with LY294002 significantly improved Bax and decreased the anti‐apoptotic protein Bcl2(Figure 6B). The opposite results were observed in cells pretreated with 740Y‐P (Figure 6D). In addition, the EdU and clone formation experiments were conducted, indicating that LY294002 and 740Y‐P significantly inhibited or enhanced the proliferation ability of the Que + shP4HA2 group cells, respectively, consistent with previous results (Figure 5A,E). The TUNEL staining showed that LY294002 substantially enhanced the quantity of apoptotic cells induced by quercetin, and the pro‐apoptotic effect of quercetin was observed in 740Y‐P (Figure 5B). Quercetin prompts cell apoptosis by downregulating P4HA2 and restraining the PI3K/Akt/mTOR pathway (Figure 7).

## 4 Discussion

Liver cancer is the leading cause of cancer‐related death worldwide [5]. HCC accounts for 85%–95% of primary liver cancer [48]. Due to the lack of clinical features, early diagnosis, and treatment, HCC patients have lower survival rates and poorer quality of life; it has become a global issue [49, 50, 51], and There is a need to discover and explore new therapeutic drugs or methods to treat HCC. In recent years, some natural compounds discovered and extracted from plants have been proven to have enormous anti‐tumor potential [39]. Quercetin has captivated much attention as a result of its beneficial effects, anti‐inflammatory, antioxidant, immune regulation, and cardiovascular protection. It has high biological activity and water solubility [28, 29, 32], which makes it effective and safe in the clinical treatment of numerous malignant tumors [33]. Yamada et al. found that quercetin inhibits HCC cell migration induced by growth factor‐α by suppressing the AKT signaling pathway [52]. Wu et al. proved that quercetin exhibits anti‐tumor effects in HCC LM3 cells by blocking the JAK2/STAT3 signaling pathway [53]. Furthermore, Utilizing nanocarriers to enhance the bioavailability and targeting of quercetin, thereby improving its therapeutic effect on HCC and overcoming resistance to anticancer drugs. When combined with traditional chemotherapy, it provides a synergistic effect to improve the prognosis of patients [53]. Nevertheless, the specific mechanism of the anti‐tumor effect of quercetin in HCC cells is still unclear. This research confirmed that quercetin obstructs cell growth and compels SNU449 and Hep‐3B cell apoptosis, playing an anti‐tumor role in HCC.

P4HA2 has played a pivotal role in the growth of tumor cells [54]. P4HA2 governs cellular biological functions in certain tumor cells, including cell proliferation and differentiation. Knocking out tumor cells P4HA2 results in cell apoptosis and growth restriction [19, 55]. Additionally, P4HA2 is a crucial enzyme in collagen synthesis that can promote collagen expression and deposition, leading to tumor growth and metastasis [18]. The downregulation of P4HA2 may impair the EMT process, leading to inhibiting their growth and metastasis [17]. Our previous study demonstrated that downregulated P4HA2 in SNU49 and Hep‐3B cells notably constrained cell growth and metastasis [46]. The anti‐tumor impact of quercetin may be associated with P4HA2. This experiment reveals that quercetin considerably lessens the expression of P4HA2 protein and mRNA. To further verify the indispensable role of P4HA2 in the action of quercetin treatment in HCC, this study downregulated the P4HA2 gene using lentiviral vectors. Subsequently, the results point out that when P4HA2 expression was downregulated, the inhibitory effect of quercetin on HCC cell proliferation was enhanced, and the promoting effect on HCC cell apoptosis was increased. P4HA2 is engaged in the anti‐HCC effect of quercetin. In summary, quercetin blocks the proliferation and fosters apoptosis of HCC cells through repressing P4HA2.

The PI3K/Akt/mTOR pathway is an essential intracellular signaling pathway that modulates various cellular activities such as cell activity, proliferation, autophagy, and growth [47, 56, 57]. Targeted apoptosis and autophagy have been recognized as potential strategies for cancer treatment [58]. The dysregulation of this pathway involves various functional impairments or diseases, including cancer [59]. For example, Plumbagin can inhibit cancer cells growth via instigating PI3K/Akt/mTOR‐intervened G2/M phase cell arrest, apoptosis, and autophagy [60]. In addition, research has found that quercetin can induce the P3K/AKT pathway and promote the death of docetaxel‐resistant cells [43]. Based on these studies, as the expression levels of phosphorylated PI3K, AKT, and mTOR decrease, the PI3K/Akt/mTOR pathway in quercetin‐treated SNU‐449 and Hep‐3B cells may be inhibited. When LY294002 pretreated shP4HA2 HCC cells, this inhibition can be enhanced with a diminish in tumor cells and an expand in cell apoptosis. Additionally, the pretreatment of 740Y‐P can remarkably overturn the effect of quercetin on inhibiting the PI3K/Akt/mTOR pathway, which can enhance tumor cell proliferation and inhibit cell apoptosis. In summary, quercetin downregulates P4HA2‐induced cell apoptosis and inhibits cell growth mediated by inhibiting the PI3K/Akt/mTOR pathway.

Apoptosis is an active programmed cell death mechanism that participates in maintaining the balance of the intracellular environment. Its characteristics include cell contraction, cytoplasmic density, and pyknosis [61], regulated by Bcl2 family proteins [62]. Drugs like BH3 [63] and many novel compounds^64^ can initiate cell apoptosis through different mechanisms. Quercetin activates cell apoptosis through activating the expressions of pro‐apoptosis proteins Caspase9, PARP, and Bax and downregulating anti‐apoptotic protein Bcl2. In addition, Apoptosis inhibitors ZVF can neutralize the spirited result of quercetin on shP4HA2 cell apoptosis. The pro‐apoptotic consequence of quercetin is achieved by downregulating P4HA2. In addition, the PI3K/Akt/mTOR signaling pathways contribute to quercetin‐induced cell apoptosis. Meanwhile, LY294002 or 740Y‐P treatment of shP4HA2 can enhance or inhibit the pro‐apoptotic impact of quercetin, respectively. In the meantime, the expression of Bax, the molecular marker of apoptosis, was upregulated. In contrast, the anti‐apoptotic protein Bcl2 was downregulated, indicating that the down‐regulation of P4HA2 by quercetin can positively regulate HCC cell apoptosis through hampering the PI3K/Akt/mTOR pathway. Due to the focus of this study on the relationship between P4HA2 and PI3K/Akt/mTOR pathways in vitro, there is a lack of in vivo validation or exploration beyond the pathway level. future research in this direction will be conducted.

## 5 Conclusion

In conclusion, this study confirms that quercetin can induce apoptosis and inhibit cell proliferation of HCC cells through the PI3K/Akt/mTOR pathway, and downregulation of P4HA2 by quercetin plays an essential role in this process. Quercetin combined with chemotherapy drugs may be a future research direction. Quercetin has broad prospects as a new medicine or auxiliary strategy for the treatment of HCC.

## Author Contributions

J.Z. and J.G. conducted experiments and writing manuscripts. Y.Q., B.G., L.Y., and J.M. performed formal analysis, investigation. W.T., Y.L., and H.L. performed formal analysis and data visualization. J.Z. and W.W. performed conceptualization, project administration, funding acquisition.

## Ethics Statement

The authors have nothing to report.

## Consent

All authors have provided their consent for publication.

## Conflicts of Interest

The authors declare no conflicts of interest.

## Data Availability

The data that support the findings of this study are available from the corresponding author upon reasonable request.
